# On the Expansion of the Surface of the Brain and Its Connexion with the Development of Mind

**Published:** 1854-02

**Authors:** 

**Affiliations:** Ann. Méd. Psych.


*On the Expansion of the surface of the brain and its connexion with the development of mind.* By BAILLARGER, Ann. Méd. Psych.

(Tianslated for the Examiner.)

The author, in the 1st part of his Essay, treats of the measurements of the expansion of the cerebral convolutions. In the 2d, considers the question, whether there exists any relation between this expansion and the development of mental power.

It had been previously attempted to determine the cerebral surface by measuring the pia mater when spread out, but it has been found impossible to do this completely. Gall attempted to measure the surface by unfolding the brain, but its yielding nature and its destruction by the finger cause numerous errors. The author endeavors to avoid these by gradually and thoroughly removing the white substance, so that the hemisphere becomes reduced to a thin layer, which can be easily unfolded and moulded in gypsum. This mould can then be gradually filled with clay, whilst between the two is inserted a thin fabric, the surface of which will admit of an accurate admeasurement. By such means, he obtained the following results. The mean of the surface-expansion of five brains amounted to 1.700 cubic ctmtr. In two instances where both hemispheres were separately measured, the surface of the right, in the first, measured 764 cubic ctmtr., and that of the left 789 cubic ctmtr.; in the second, the right measured 853 cubic ctmtr., and the left 837 cubic ctmtr.

The cerebral surface of rabbits measured = 24, of cats = 52, of dogs = 104, of sheep = 160; and of swine = 220 cubic ctmtr.

As regards the relation of the surface expansion of the brain to the development of intelligence, the author observes, that both the expansion of the surface and the relative volume of the brain should be taken into consideration. For instance, the brain of the dog has less surface than that of the ox, though the dog has more intellignce.

When the compactness of the various brains is about the same, its volume is in proportion to its weight: which can be therefore, substituted for the volume. In comparing the brain of a man with that of a rabbit, it was found that the hemispheres of the former weighed, after removing the membranes, the corpora striata, the thalami optici and the corpus callosum, in all 900 grammes. Its expansion was = 1.700 cubicc tmtr. The hemispheres of the rabbit weighed 5 grammes. Its surface measured 24 ctmr. The man's brain weighed therefore, 180 times more than the rabbit: whilst its surface was only 70 times greater; therefore the hemispheres of the rabbit posseses in proportion to its weight, a surface 2½ times greater than that of man.

The author has in this manner compared the brain of man with those of rabbits, dogs, sheep and swine; and the surface of these, in connection with their weight, has always proved to be about ¼, ⅓ or ½ more than in man. The smallest brains have always the largest surface and the expansion of the hemispheres seem to bear inverse proportion to their weight. He comes therefore to the conclusion, that the development of intelligence is not in a direct but apparently in an inverse ratio to the extent of cerebral surface.—(*Schmidt's Jahrbuch.*)

